# A Polyvinyl Chloride Eraser as a Surface Marker for Computed Tomography in Emergency Imaging; a Letter to Editor

**Published:** 2019-09-28

**Authors:** Yuya Murakami, Taihei Yamada, Hiromichi Naito

**Affiliations:** 1 Department of Emergency, Critical Care and Disaster Medicine, Okayama University Hospital, Okayama, Japan.

**Keywords:** Tomography, X-Ray Computed, Emergency Service, Hospital, diagnostic imaging, radiology

**Dear Editor,**

In the wake of recent progress in computed tomography (CT) enabling to obtain high quality images within five minutes, CT scan has been widely used in emergency diagnostic radiology and is considered best suited for detection of pathologies, as well as assessing the location and extent of lesions in the emergency department. 

Emergency physicians often require confirmation of surface bruises or wounds coexisting with pathology on CT scan. Alternatively, they may want to confirm the presence or absence of pathology in the painful lesion. The use of an appropriate surface marker on the skin surface of the painful lesion may enable them to identify areas of interest and safely reduce radiation exposure. 

CT skin markers detected as virtually artifact-free and opaque, particularly for mammography, have been commercially available from several healthcare companies, but these markers are expensive ($57-$86 USD) and may not be easily accessible. Most metal objects cause artifacts through multiple mechanisms, including beam hardening, scatter, and Poisson noise, although some can be reduced using iterative reconstruction or by combining data from multiple scans (1). 

**Figure 1:**
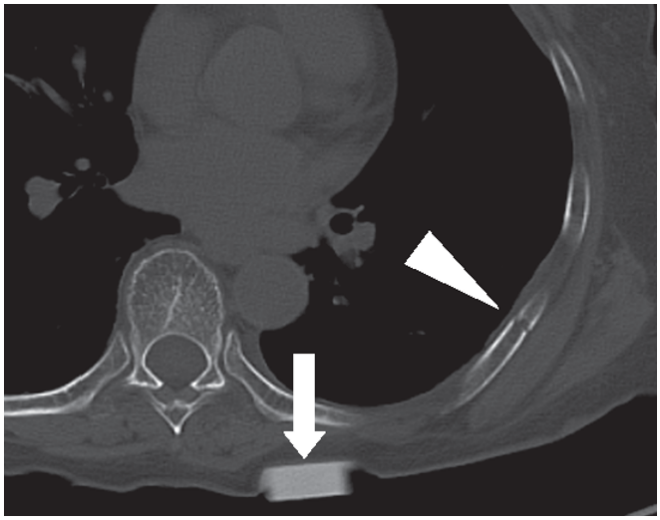
Computed tomography scan of the chest revealed correspondence of rib fracture (arrowhead) and pain location, marked with an eraser (arrow)

We found that a polyvinyl chloride eraser may be the best surface marker for CT marking applications. As shown in Figure 1, a polyvinyl chloride eraser is visualized as a high-density, artifact-free homogenous object on CT scan and can be used as a skin surface marker in the emergency setting. Interestingly, an eraser impacted in the nose of a five-year-old girl was reported to be identified as a calcified nodular mass by CT, which was diagnosed as rhinoliths. Thus, an eraser is detected as a high-density and opaque object without artifacts (2, 3).

 In conclusion, a polyvinyl chloride eraser, inexpensive and easily available in the stationery section of retail stores, even in developing countries, can serve as a suitable surface marker for CT examination in an emergency setting to localize pathology from the skin surface. This study protocol was approved by ethical committee of Okayama University Hospital.
